# Lactate Arterial-Central Venous Gradient among COVID-19 Patients in ICU: A Potential Tool in the Clinical Practice

**DOI:** 10.1155/2020/4743904

**Published:** 2020-09-25

**Authors:** Giuseppe Nardi, Gianfranco Sanson, Lucia Tassinari, Giovanna Guiotto, Antonella Potalivo, Jonathan Montomoli, Fernando Schiraldi

**Affiliations:** ^1^Dept. of Anaesthesia and Intensive Care, Infermi Hospital, Viale Settembrini 2, 47921 Rimini, Italy; ^2^Clinical Dept. of Medical, Surgical and Health Sciences, University of Trieste, Strada Di Fiume 447, 34149 Trieste, Italy; ^3^Dept of Emergency Medicine, AORN San Pio, Via Pacevecchia 53, 82100 Benevento, Italy; ^4^Emergency Dept San Paolo Hospital, Via Terracina, 80125 Naples, Italy

## Abstract

**Objective:**

In physiological conditions, arterial blood lactate concentration is equal to or lower than central venous blood lactate concentration. A reversal in this rate (i.e., higher lactate concentration in central venous blood), which could reflect a derangement in the mitochondrial metabolism of lung cells induced by inflammation, has been previously reported in patients with ARDS but has been never explored in COVID-19 patients. The aim of this study was to explore if the COVID-19-induced lung cell damage was mirrored by an arterial lactatemia higher than the central venous one; then if the administration of anti-inflammatory therapy (i.e., canakinumab 300 mg subcutaneous) could normalize such abnormal lactate a-cv difference.

**Methods:**

A prospective cohort study was conducted, started on March 25, 2020, for a duration of 10 days, enrolling 21 patients affected by severe COVID-19 pneumonia undergoing mechanical ventilation consecutively admitted to the ICU of the Rimini Hospital, Italy. Arterial and central venous blood samples were contemporarily collected to calculate the difference between arterial and central venous lactate (Delta a-cv lactate) concentrations within 24 h from tracheal intubation (*T*_0_) and 24 hours after canakinumab administration (*T*_1_).

**Results:**

At *T*_0_, 19 of 21 (90.5%) patients showed a pathologic Delta a-cv lactate (median 0.15 mmol/L; IQR 0.07–0.25). In the 13 patients undergoing canakinumab administration, at *T*_1_, Delta a-cv lactate decreased in 92.3% of cases, the decrease being statistically significant (*T*_0_: median 0.24, IQR 0.09–0.31 mmol/L; *T*_1_: median −0.01, IQR −0.08–0.04 mmol/L; *p*=0.002).

**Conclusion:**

A reversed Delta a-cv lactate might be interpreted as one of the effects of COVID-19-related cytokine storm, which could reflect a derangement in the mitochondrial metabolism of lung cells induced by severe inflammation or other uncoupling mediators. In addition, Delta a-cv lactate decrease might also reflect the anti-inflammatory activity of canakinumab. Our preliminary findings need to be confirmed by larger outcome studies.

## 1. Background

The role of serial lactate determinations in critically ill patients is well agreed and may be useful in tailoring the therapy in many different diseases [1–3]. There is consensus about the two mainly observed forms of raised blood lactate concentration: lactic acidosis due to O_2_-demand/DO_2_ mismatch [4] and hyperlactatemia with near-normal arterial pH, the latter being substantially linked to hypermetabolic stress, or inherited disease [5].

In physiological conditions, arterial blood lactate concentration is equal to or lower than central venous blood lactate concentration (Delta a-cv lactate, normal value ≤ 0 mmol/L) [6]. Less is known about the meaning of a Delta a-cv lactate reversal (a-cv difference > 0 mmol/L), which could reflect a derangement in the mitochondrial metabolism of lung cells induced by inflammation or other uncoupling mediators, likely to be responsible for the large lung parenchymal disruption (acute respiratory distress syndrome- (ARDS-) like) [7]. Indeed, De Backer et al. [8] demonstrated that in ARDS the lungs produce increased amounts of lactate, so that the arterial lactate concentration is greater than the central venous one, even if the arterial concentration is still normal. In a similar study, Kellum et al. [9] confirmed the hypothesis demonstrating that the abnormal lactate a-cv difference was strongly correlated with the lung injury score. To our knowledge, nobody has reported such an abnormal a-cv lactate difference in coronavirus disease 2019 (COVID-19) patients.

Patients affected by severe COVID-19 pneumonia usually present with the first phase of disease characterized by acute viremia (fever, cough, and myalgia), followed by the second one, where lung injury is mainly caused by severe inflammation due to a massive release of cytokines (“cytokine storm”) [10, 11]. Administration of anti-inflammatory drugs (immunosuppressive monoclonal antibodies) such as tocilizumab and canakinumab has been proposed to limit the “storm effect” and therefore reduce lung damage [12]. Canakinumab is a monoclonal antibody targeted at interleukin-1*β* (IL-1*β*) and so far its clinical use has been limited to treatment of the juvenile idiopathic arthritis and other autoinflammatory conditions [13–16]. Our hypothesis is that the lung injury could be well depicted by an abnormal lactate a-cv gradient, while the anti-inflammatory effect of canakinumab, if any, could be mirrored by a reduction of that parameter. To our knowledge, no previous studies have examined the correlation between the lactate a-cv gradient and the use of canakinumab.

Therefore, we performed the present study with the following aims: to describe the Delta a-cv lactate in a population of patients affected by severe COVID-19 pneumonia (early warning tool) and to evaluate if the reduction/normalization of the Delta a-cv lactate was positively affected by canakinumab administration (early response tool).

## 2. Methods

### 2.1. Study Design, Setting, and Population

This is a preliminary report of the CANASCOV study (CANAkinumab Study on COronaVirus pneumonia). The study was conducted in the intensive care unit (ICU) of the Infermi Hospital, Rimini, Italy, started on March 25, 2020, and ended on April 3, 2020, for a total duration of 10 days.

All adult (age ≥ 18 years) patients consecutively admitted to the ICU with severe COVID-19 pneumonia who required sedation and invasive mechanical ventilation were considered for inclusion. In the event that the baseline coupled arterial-central venous lactate sampling was not obtained within 24 h from endotracheal intubation, the patient was excluded. Suspected or confirmed bacterial infection (ventilator-associated pneumonia; urinary tract infection; positive blood culture; white blood cells > 12.000; procalcitonin >1), renal failure (estimated glomerular filtrate rate < 60 mL/min/1.73 m^2^) [17], and impaired liver function (liver enzyme increase more than five times the normal values) were considered contraindications to administration of canakinumab, and therefore, patients with any of the previous conditions contributed only with the baseline measurement of the Delta a-cv lactate.

### 2.2. Data Collection

At ICU admission, general patient demographic information was collected and the Simplified Acute Physiology Score (SAPS II) [18] and the Brixia score [19] were computed. The Brixia score is a method developed to assess the extent of lung damage in COVID-19 patients from the chest radiogram. Briefly, the lungs are divided into three sectors on the transverse plane; to each of the obtained six sectors, a score is assigned, ranging from 0 (no alteration) to 3 (interstitial-alveolar infiltrates). The score assigned to each sector is summed up and the overall score obtained may range from 0 to 18.

All patients were treated with hydroxychloroquine (Plaquenil^®^, Sanofi, 200 mg twice a day). Steroids were prescribed only after 7 days from hospital admission to avoid administration of immunosuppressive drugs during the acute viremic phase. Administration of steroids and antiviral drugs was also documented.

Within 24 h after tracheal intubation, one arterial blood sample and one central venous blood sample were contemporarily collected by two operators through an arterial catheter and a central venous catheter with the distal tip placed in the lower third of the superior vena cava, respectively. Both samples were analyzed with an Automatic QC Gas Analyzer (Siemens, Munich, Germany), and the difference between arterial and central venous lactate (Delta a-cv lactate) concentrations was calculated. PaO_2_ and both arterial and central venous PCO_2_ were also measured, and PaO_2_/FiO_2_ ratio and Delta cv-a PCO_2_ were determined. Serum interleukin-6 (IL-6, pg/ml), creatinine (mg/dL), and alanine aminotransferase (ALT, U/L) levels were also measured at the same time. Since the laboratory simply reported IL-6 as “>1000” when its concentration exceeded this threshold, a concentration of 1000 pg/ml was considered for analysis purposes in these cases. Canakinumab (Ilaris^®^, Novartis) 300 mg was administered by subcutaneous route in patients submitted to mechanical ventilation because of severe pneumonia caused by COVID-19. Delta a-cv lactate was reassessed 24 h after drug administration in patients treated with canakinumab.

### 2.3. Ethical Considerations

Daily arterial and central venous blood samplings are part of the standard clinical practice for patient's hemodynamic and oxyphoretic assessment. Therefore, no blood sampling was performed specifically for the study purpose. The current study was conducted according to the ethical principles of the Declaration of Helsinki and was approved by the Hospital Ethical Board (registration number NCT04348448). The research did not affect any clinical decision making in a patient's care.

### 2.4. Data Analysis

The data distribution was evaluated using the Kolmogorov–Smirnov test; all variables but SAPS II, Brixia score, and PaO_2_/FiO_2_ ratio showed a skewed distribution. The nominal variables were described as numbers and percentages and the continuous variables as medians and interquartile ranges (IQRs). Unadjusted comparisons between groups were investigated through Fisher's exact test, Mann–Whitney *U* test for independent samples, or Wilcoxon rank for matched samples, as appropriate. Bivariate association between Delta a-cv lactate and the other study variables was investigated with parametric Pearson's (*r*) or nonparametric Spearman's (*ρ*) correlation coefficient, as appropriate. Positive or negative correlation strengths were interpreted as follows: 0–0.30: negligible; 0.30–0.50: low; 0.50–0.70: moderate; 0.70–0.90: high; and 0.90–1: very high [20]. For all tests, statistical significance was set at an alpha level of *p*=0.05. All statistical analyses were performed using the software IBM SPSS Statistics, version 24.0 (Armonk, NY, US: IBM Corp).

## 3. Results

During the study period, 23 patients suffering from COVID-19-related pneumonia were admitted to the ICU. All patients but two were submitted to a synchronous coupled arterial and central venous sampling for blood gas analysis. Overall, the study population was constituted by 21 patients (18 males, 85.7%), whose main baseline characteristics and outcomes at ICU discharge are described in Table 1.

The median Delta a-cv lactate on admission was 0.15 mmol/L (IQR: 0.07–0.25) in the overall population. Among the 21 patients, 19 (90.5%) showed an inverted Delta a-cv lactate (higher lactate level in the arterial than in central venous blood) ranging between 0.05 and 0.51 mmol/L. No statistically significant difference was shown in Delta a-cv lactate after stratification of patients according to patients' sex and administrations of steroids and antiviral drugs. Delta a-cv lactate showed a statistically significant moderate negative correlation with Delta cv-a PCO_2_ (*r* = −0.528; *p*=0.014), whilst no further significant correlations were found with severity scores, demographic, comorbidity, and laboratory variables (Table 2).

**Figure 1:**
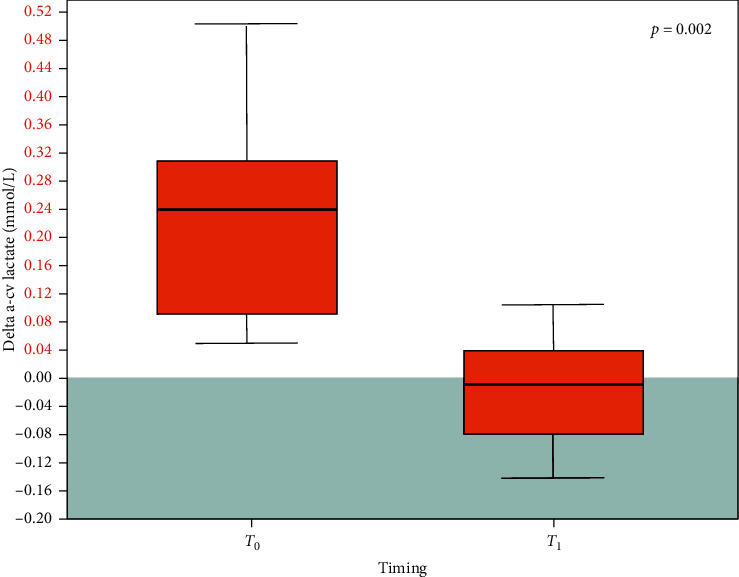
Delta a-cv lactate before (*T*_0_) and after (*T*_1_) canakinumab administration in 13 patients. Black horizontal line inside the boxes: median. Box height: interquartile range. Grey area: normal Delta a-cv lactate values (≤0).

Thirteen patients (61.9%) were treated with canakinumab. After the treatment, Delta a-cv lactate decreased in all but one patient (92.3%), with eight out of 13 patients (61.5%) presenting a normalization of Delta a-cv lactate (Table 3). Delta a-cv lactate decrease between *T*_0_ and *T*_1_ was statistically significant (*T*_0_: median 0.24, IQR 0.09–0.31 mmol/L; *T*_1_: median −0.01, IQR −0.08–0.04 mmol/L; *p*=0.002; Figure 1).

## 4. Discussion

The study findings suggest that patients admitted to the ICU with COVID-19 pneumonia are likely to have an inverted Delta a-cv lactate that may be an early marker of the ongoing biological damage mediated by the virus-related inflammation. To the best of our knowledge, this is the first study considering Delta a-cv lactate assessment in mechanically ventilated ICU patients with severe COVID-19 pneumonia.

Interestingly, an increase in the Delta cv-a PCO_2_ was not observed in the study population, as might have been expected if the Delta a-cv lactate increase had been attributable to a systemic increase in arterial lactate production as in the case of an anaerobic metabolism due to severe tissue hypoxia. Indeed, Delta cv-a PCO_2_ could increase significantly in conditions of low cardiac input and hypoperfusion [21], but this was not the case of our patients who presented normal/near-normal arterial lactate levels, confirming a substantial total body adequacy of perfusion. These findings may support our hypothesis for a pulmonary overproduction of lactate, which is likely to be considered as a lung “inflammatory” marker not linked to any cardiac output inadequacy.

Moreover, our data documented a significant improvement in the Delta a-cv lactate in the majority of patients treated with canakinumab. This finding could be interpreted as a favorable, early effect of the administered medication, supporting the hypothesis of a reduction of cytokine-related severe inflammation at the lung level. However, other results such as the lack of a linear correlation between the Delta a-cv lactate and IL-6 levels require caution with the interpretation of the study results, in particular about the role of immunosuppressive drugs in the treatment of the COVID-19. Indeed, the use of immunosuppressive monoclonal antibodies is associated with potential risks of liver injury and decreased patient's immunocompetence. Therefore, more data and a larger sample of patients are needed to provide a better understanding of therapeutic mechanisms and to analyze in deep the effectiveness of these anti-inflammatory drugs.

As the COVID outbreak is becoming a real “stress test” for healthcare systems all over the world, many different pharmacologic strategies are under investigation against strong outcome indicators, such as in-hospital death or ICU length of stay. In this context, the availability of predictors able to identify early, quickly, and without additional costs patients in whom the infection is having a greater impact is pivotal. Interestingly, Delta a-cv lactate data can be collected routinely bedside and almost for free, even in an overwhelmed ICU context, as the COVID-19 patients in ICU are usually submitted to central venous and arterial cannulation to allow hemodynamic monitoring and blood gas analysis.

## 5. Limitations

Our preliminary report has several methodologic limitations that should be considered when interpreting our results. The study had an observational design with descriptive and correlational aims and enrolled a rather small sample in a single center. Thus, the role of Delta a-cv lactate as an early marker of severe lung inflammation in COVID-19 patients should be confirmed by further studies.

Although our findings may be explained by the effect of the administered drug, a direct comparison of the trend of the Delta a-cv lactate in a group of patients receiving the standard of care was hampered by the lack of a control group. Therefore, the possible causative relationship between canakinumab administration and Delta a-cv lactate decrease, as well as the predictive power of these variables on patients' key outcomes, should be examined in randomized clinical studies with larger populations and considering relevant confounders.

## 6. Conclusion

The serial evaluation of Delta a-cv lactate trends seems to be a fast reliable tool to get a further insight about the lung biological disarray, perhaps even useful to better tailor the therapy in COVID patients. Nevertheless, our data can only suggest that the use of canakinumab may normalize the Delta a-cv lactate, thus supporting the hypothesis of a reduction of inflammation at the lung level. Therefore, our results should be only considered as a contribution to a better understanding of the physiopathology of COVID-19-induced lung injury.

## Figures and Tables

**Table 1:** Baseline characteristics on ICU admission and ICU outcomes of the enrolled population.

Variable	Median (IQR)
Age (years)	66.0 (55.0–71.0)
Charlson comorbidity index	2.0 (1.0–3.0)
SAPS II score	37.0 (29.0–41.5)
Brixia score	10.0 (9.0–11.5)
Interleukin-6 (pg/mL)	40.3 (10.0–759.5)
Alanine aminotransferase (U/L)	32.0 (20.5–83.5)
Creatinine (mg/dL)	0.75 (0.58–0.87)
PaO_2_/FiO_2_ ratio	195.0 (152.5–219.5)
PaCO_2_ (mmHg)	51.7 (41.6–55.6)
Arterial lactate (mmol/L)	1.65 (0.96–2.07)
Central venous lactate (mmol/L)	1.44 (0.76–1.84)
Delta a-cv lactate (mmol/L)	0.15 (0.07–0.25)
Length of mechanical ventilation (days)	19.0 (10.0–42.0)
Length of stay in ICU (days)	22.0 (12.50–49.50)
Survival rate at ICU discharge (*n*; %)	16; 76.2%

ICU: intensive care unit; IQR: interquartile range; SAPS II: Simplified Acute Physiology Score; a-cv: arterial-central venous.

**Table 2:** Results of the correlation analyses between Delta a-cv lactate and the study variables.

Variable	Correlation
Age (years)	*ρ* = −0.278 (*p*=0.223)
Charlson comorbidity index	*ρ* = −0.163 (*p*=0.480)
SAPS II score	*r* = −0.009 (*p*=0.968)
Brixia score	*r* = −0.161 (*p*=0.485)
Interleukin-6 (pg/mL)	*ρ* = 0.203 (*p*=0.377)
Alanine aminotransferase (U/L)	*ρ* = 0.111 (*p*=0.632)
Creatinine (mg/dL)	*ρ* = −0.056 (*p*=0.811)
PaO_2_/FiO_2_ ratio	*r* = 0.182 (*p*=0.430)
Delta cv-a PCO_2_ (mmHg)	*ρ* = −0.528 (*p*=0.014)

*r*-value: Pearson's correlation coefficient; *ρ*-value: Spearman's correlation coefficient; SAPS II: Simplified Acute Physiology Score; a-cv: arterial-central venous; cv-a: central venous-arterial.

**Table 3:** Main characteristics and Delta a-cv lactate variation in 13 patients treated with canakinumab.

Sex	Age (years)	PaO_2_/FiO_2_ ratio	Brixia score	IL-6 (pg/mL)	Delta a-cv lactate		
					*T*_0_	*T*_1_	*T*_0_–*T*_1_
Male	32	218	10	14	0.26	−0.77	−1.03
Male	64	195	12	>1000	0.41	−0.11	−0.52
Male	54	221	9	>1000	0.51	0.02	−0.49
Male	59	229	8	47	0.24	−0.14	−0.38
Male	72	203	11	>1000	0.24	−0.03	−0.27
Male	64	231	11	519	0.19	−0.06	−0.25
Male	67	146	9	14	0.24	0.05	−0.19
Male	65	149	6	20	0.15	−0.01	−0.16
Male	55	156	12	10	0.36	0.23	−0.13
Male	69	204	6	7	0.11	0.01	−0.10
Male	54	203	10	292	0.07	−0.03	−0.10
Male	67	166	11	35	0.06	0.00	−0.06
Male	66	183	9	5	0.05	0.11	0.06

IL-6: interleukin-6; *T*_0_: before canakinumab administration; *T*_1_: after canakinumab administration.

## Data Availability

The data used to support the study results are available on request.

## References

[1] Bakker J., Gris P., Coffernils M., Kahn R. J., Vincent J.-L. (1996). Serial blood lactate levels can predict the development of multiple organ failure following septic shock. *The American Journal of Surgery*.

[2] Correa T. D., Pereira A. J., Brandt S. (2017). Time course of blood lactate levels, inflammation, and mitochondrial function in experimental sepsis. *Critical Care*.

[3] Scott S., Antonaglia V., Guiotto G., Paladino F., Schiraldi F. (2010). Two-hour lactate clearance predicts negative outcome in patients with cardiorespiratory insufficiency. *Critical Care Research and Practice*.

[4] Routsi C., Vincent J. L., Bakker J. (1993). Relation between oxygen consumption and oxygen delivery in patients after cardiac surgery. *Anesthesia and Analgesia*.

[5] Grip J., Falkenström T., Promsin P., Wernerman J., Norberg Å., Rooyackers O. (2020). Lactate kinetics in ICU patients using a bolus of 13 C-labeled lactate. *Critical Care*.

[6] Zhou X., Ye Y., Tian F., Wu F. (2017). Lactate levels in arterial and venous blood may be correlated but not equivalent. *Journal of Critical Care*.

[7] Viswan A., Ghosh P., Gupta D., Azim A., Sinha N. (2019). Distinct metabolic endotype mirroring acute respiratory distress syndrome (ARDS) subphenotype and its heterogeneous biology. *Scientific Reports*.

[8] De Backer D., Creteur J., Zhang H., Norrenberg M., Vincent J. L. (1997). Lactate production by the lungs in acute lung injury. *American Journal of Respiratory and Critical Care Medicine*.

[9] Kellum J. A., Kramer D. J., Lee K., Mankad S., Bellomo R., Pinsky M. R. (1997). Release of lactate by the lung in acute lung injury. *Chest*.

[10] Tang X., Du R., Wang R. (2020). Comparison of hospitalized patients with acute respiratory distress syndrome caused by COVID-19 and H1N1. *Chest*.

[11] Tisoncik J. R., Korth M. J., Simmons C. P., Farrar J., Martin T. R., Katze M. G. (2012). Into the eye of the cytokine storm. *Microbiology and Molecular Biology Reviews*.

[12] Zhang W., Zhao Y., Zhang F. (2020). The use of anti-inflammatory drugs in the treatment of people with severe coronavirus disease 2019 (COVID-19): the perspectives of clinical immunologists from China. *Clinical Immunology*.

[13] De Benedetti F., Gattorno M., Anton J. (2018). Canakinumab for the treatment of autoinflammatory recurrent fever syndromes. *New England Journal of Medicine*.

[14] Everett B. M., Donath M. Y., Pradhan A. D. (2018). Anti-Inflammatory therapy with canakinumab for the prevention and management of diabetes. *Journal of the American College of Cardiology*.

[15] Ridker P. M., Everett B. M., Thuren T. (2017). Antiinflammatory therapy with canakinumab for atherosclerotic disease. *New England Journal of Medicine*.

[16] Ruperto N., Brunner H. I., Quartier P. (2018). Canakinumab in patients with systemic juvenile idiopathic arthritis and active systemic features: results from the 5-year long-term extension of the phase III pivotal trials. *Annals of the Rheumatic Diseases*.

[17] Levey A. S., Coresh J., Balk E. (2003). National Kidney Foundation practice guidelines for chronic kidney disease: evaluation, classification, and stratification. *Annals of Internal Medicine*.

[18] Le Gall J. R., Lemeshow S., Saulnier F. (1993). A new Simplified Acute Physiology Score (SAPS II) based on a European/North American multicenter study. *JAMA: The Journal of the American Medical Association*.

[19] Borghesi A., Zigliani A., Masciullo R. (2020). Radiographic severity index in COVID-19 pneumonia: relationship to age and sex in 783 Italian patients. *Research Square*.

[20] Hinkle D. E., Wiersma W., Jurs S. G. (2003). *Applied Statistics for the Behavioral Sciences*.

[21] Lamia B., Monnet X., Teboul J. L. (2006). Meaning of arterio-venous PCO_2_ difference in circulatory shock. *Minerva Anestesiologica*.

